# Human Hsp60 with Its Mitochondrial Import Signal Occurs in Solution as Heptamers and Tetradecamers Remarkably Stable over a Wide Range of Concentrations

**DOI:** 10.1371/journal.pone.0097657

**Published:** 2014-05-15

**Authors:** Silvia Vilasi, Rita Carrotta, Maria Rosalia Mangione, Claudia Campanella, Fabio Librizzi, Loredana Randazzo, Vincenzo Martorana, Antonella Marino Gammazza, Maria Grazia Ortore, Annalisa Vilasi, Gabriella Pocsfalvi, Giosalba Burgio, Davide Corona, Antonio Palumbo Piccionello, Giovanni Zummo, Donatella Bulone, Everly Conway de Macario, Alberto J. L. Macario, Pier Luigi San Biagio, Francesco Cappello

**Affiliations:** 1 Institute of Biophysics, National Research Council, Palermo, Italy; 2 Department of Experimental Biomedicine and Clinical Neurosciences, University of Palermo, Palermo, Italy; 3 Euro-Mediterranean Institute of Science and Technology, Palermo, Italy; 4 Department of Life and Environmental Sciences and National Interuniversity Consortium for the Physical Sciences of Matter, Marche Polytechnic University, Ancona, Italy; 5 Institute of Biosciences and Bioresources, National Research Council, Napoli, Italy; 6 Department of biological chemical and pharmaceutical sciences and technologies, University of Palermo, Palermo, Italy; 7 Department of Microbiology and Immunology, School of Medicine, University of Maryland at Baltimore, and Institute of Marine and Environmental Technology, Columbus Center, Baltimore, Maryland, United States of America; National Institute for Medical Research, Medical Research Council, London, United Kingdom

## Abstract

It has been established that Hsp60 can accumulate in the cytosol in various pathological conditions, including cancer and chronic inflammatory diseases. Part or all of the cytosolic Hsp60 could be naïve, namely, bear the mitochondrial import signal (MIS), but neither the structure nor the in solution oligomeric organization of this cytosolic molecule has still been elucidated. Here we present a detailed study of the structure and self-organization of naïve cytosolic Hsp60 in solution. Results were obtained by different biophysical methods (light and X ray scattering, single molecule spectroscopy and hydrodynamics) that all together allowed us to assay a wide range of concentrations of Hsp60. We found that Naïve Hsp60 in aqueous solution is assembled in very stable heptamers and tetradecamers at all concentrations assayed, without any trace of monomer presence.

## Introduction

Hsp60 is a molecular chaperone, highly conserved during evolution that assists protein folding in mitochondria [1], [2]. It is encoded and transcribed by a nuclear gene and translated in the cytosol. The newly translated (“naïve”) polypeptide has a mitochondrial import signal (MIS), i.e., a sequence of 26 amino acids at the N-terminus that drives Hsp60 to the inside of mitochondria where the MIS is cleaved and the protein reaches the final conformation (mtHsp60 or Cpn60) [3]. A peptide derived from the signal sequence MIS of human Hsp60 has been found to be present in human histocompatibility leukocyte antigen (HLA)-E and to be involved in the detection mechanism of stressed cells [4].

The mitochondrial import of Hsp60, similarly to that of other protein precursors, is a complex mechanism that depends on the mitochondrial membrane potential, and involves several molecular chaperones, such as Hsp70, in the matrix space [5], [6] as well as in the cytosol [7], [8]. Once Hsp60 is imported into mitochondria, its folding and self-assembly from monomers to oligomeric species is mediated by functional pre-existing Hsp60 complexes that catalyse chaperonin folding in an ATP-dependent process [9].

In the yeast mitochondrion, analogously to the bacterial homolog GroEL, mtHsp60 self-assembles in ring-shaped heptameric quaternary structure, two of which associate to form a barrel-shaped tetradecamer, which is the ATP-driven functional macromolecular chaperoning complex [9]–[12]. Hsp60 has been found in the mitochondria of a variety of eukaryotic cells, including human cell lines [13]–[15]. However, differently from bacterial homologs that exist only as tetradecamers, the human mtHsp60 seems to exist as a homo-oligomer of seven subunits [13]. Moreover, *in vitro* studies have shown that some mammalian mtHsp60, including those from human and Chinese hamster cells, when purified as recombinants from *E*. *coli*, occur mainly as heptameric rings, in equilibrium with very minor populations of monomers and double-ring tetradecamers [16]–[18] and a single ring seems to be sufficient for productive chaperonin-mediated folding *in vivo* [19]. The “two-stroke engine” has been demonstrated to be necessary for productive facilitated folding in the double toroidal structure GroEL/GroES because the co-chaperonin GroES release is induced by the transfer of allosteric information between the two rings [20], [21]. Instead, mtHsp60 can function as an efficient “one stroke engine”, due to the very lower affinity of Hsp60 to its co-chaperonin Hsp10 in respect to the binding parameters characterizing the GroEL/GroES interaction [19]. Moreover, two of the four residues (R452, E461, S463 and V464) that are essential for the GroEL double ring formation, are different in the corresponding Hsp60 positions [19]. Only in the presence of ATP and/or its own 10 kDa co-chaperonin Hsp10, the mtHsp60 heptamers are able to dimerize, and to form double rings [16], [22], [23]. *In vitro*, the protein is unstable and can rapidly dissociate into monomers if incubated at 3.3 µM concentration at low temperatures (e.g., 4°C) or in the presence of ATP [17], [24]. ATP seems to play a dual role: it is necessary for mtHsp60 oligomerization at higher protein concentrations (80.2 µM) and favors dissociation into monomers al lower concentrations (3.3 µM) [24].

Very recently, the solid state structure of the mammalian mitochondrial Hsp60-Hsp10 complex has been determined for the first time by X-ray diffraction methods. The structure appeared as a symmetrical ‘football’-shaped complex of the chaperonin with the co-chaperonin [25].

It has been shown that Hsp60 plays key roles outside mitochondria, too [6], [26]–[31]. In pathologic situations, such as cancer and autoimmune/inflammatory diseases, Hsp60 accumulates in the cytosol as demonstrated by various techniques [26], [27]. From the cytosol, Hsp60 may reach other cellular compartments, such as the Golgi, secretory vesicles, and plasma membrane in normal [32], [33] and tumor [34], [35] cells. Each one of these locations may have specific implications for pathogenesis and disease progression. For example, Hsp60 accumulating in the cytosol of tumor cells can prevent pro-caspase-3 activation, in turn blocking apoptosis [28] and, for this reason, Hsp60 has been proposed as a good candidate target for anti-cancer therapy [36], [37].

The most accepted hypothesis is that cytosolic accumulation of Hsp60 could occur via a complex mitochondrial export mechanism [6], but nothing in this regard has ever been demonstrated in the case of Hsp60 cytosolic increase detected in tumor cells or during inflammatory diseases [26], [27]. Rather, there is evidence that, in some cases, Hsp60 can reside and accumulate in the cytosol without being imported into mitochondria after its synthesis, and, therefore bearing the signal sequence MIS [28] [8]. An example is provided by LNCaP cellular systems in which the exposition to specific apoptosis inducers, such as serum starvation or Dox treatment, causes Hsp60 accumulation without involving apparent mitochondrial release [28]. Moreover, an antibody against the signal sequence of Hsp60 was cross-reacted with a protein that is stably present only in the cytoplasm of rat liver [8] and, even if not explicitly commented, two bands are present in the western blot (with anti-HSP60 monoclonal antibody) of intact cytoplasm fraction from adult cardiac myocytes [38]. Also, the cytosolic Hsp60 accumulation mechanisms may occur with or without mitochondrial release concomitantly [28], so that in the cytosol the two types of 60 kDa chaperonin proteins, mtHsp60 and its precursor naïve form, could coexist.

While several studies have been performed to assess stability, oligomeric structure, and folding activity of purified mammalian mtHsp60 [16]–[19], [24], little is known about the chaperonin, possibly naïve that, as said above, could accumulate in the cytosol with its MIS. From studies on GroEL structure [39] and GroEL mutants [40] we can assume that the presequence fits inside the naïve Hsp60 cavity. However, this occurrence was not directly detected, neither it is known whether the additional residues are able to influence the protein oligomeric structure. Moreover, there are few reports concerning the import system of Hsp60 into mitochondria. Besides the essential requirement of the N-terminal sequence [6], it has been proven that the import mechanism involves the cytoplasmatic Hsp70, presumably by keeping the protein as a monomer [8].

But, what happens to the Hsp60 that could accumulate in the cytosol without entering the mitochondria? What is its oligomeric state? Is it able to form oligomeric complexes, such as heptamers or tetradecamers, which for GroEL and mtHsp60 are considered the functional chaperonin forms? Shedding light on naïve Hsp60 structure and oligomeric state *in vitro* could help to validate its role in all cases, physiological or pathological, in which it is not known if the protein accumulates in the cytosol in its native or mature form.

In this study, we addressed some of these unresolved issues concerning the structural characteristics and oligomeric state of the cytosolic Hsp60. In order to pursue this goal, we investigated the oligomeric state and stability of Hsp60 naïve *in vitro* by a battery of biophysical methods applied over a concentration range spanning from 10 nM to 79.5 µM. This range is wide enough to be supposed to cover the Hsp60 concentrations found *in vivo* under physiological conditions, and during the progression of diseases characterized by an increase of the chaperonin in the affected tissues, as determined by semi-quantitative immunohistochemistry.

Even if there are no direct measurements on the Hsp60 concentrations in the cytosol and mitochondria in normal or pathologic conditions, it is known that Hsp60 levels in human blood occurs with a wide range of concentrations, from 0.02 nM to 12.6 µM [41], [42].

The approach used in this study, based on biophysical methodologies, aimed to investigate the oligomeric structure and stability of naïve Hsp60 under cell-free conditions. This allowed us to elucidate the structural basis underlying Hsp60 functions, thus providing information relevant to draw what may occur inside cells in the cases in which the precursor form of the protein accumulates in the cytosol, both at physiological and pathological levels.

## Material and Methods

### ATPase activity of recombinant proteins

As a step prior to the experiments, we verified that the chaperonins under analysis were able to hydrolyze ATP (Figure S1). The recombinant naïve Hsp60 was obtained from ATGen (Seongnam, South Korea) in stock solution at 16.0 µM (1 mg/ml) (buffer 20 mM Tris pH 8.0 and 10% glycerol (w/w)). Lyophilized GroEL was obtained from SIGMA (St. Louis, MO, USA). ATPase assay was performed as previously described [43]. Briefly, recombinant naïve Hsp60 or GroEL was added to ATPase buffer (6.6 mM HEPES (pH 7.6), 0.66 mM EDTA, 0.66 mM 2-mercaptoethanol, 0.033% NP-40, 1.1 mM MgCl_2_, 33 µM ATP, 5 µCi (γ-33P) ATP-3000 mmol^−1^ (Ge Healthcare)), and 100 ng of plasmidic DNA was used as substrate. The buffer was used as control and it was setup in parallel. The reactions were incubated for 30 min at 24°C. Unreacted ATP and free y-phosphate were separated by thin layer chromatography, using TLC cellulose (Merck Millipore, Milan, Italy). ATP hydrolysis quantification was done with a Bio-Rad (Berkeley, CA, USA) Personal Molecular Imager FX System.

### Sample preparation for biophysics experiments

All the experiments were conducted by diluting naïve Hsp60 from stock solutions to reach the concentration of 4.8 µM and dissolving GroEL in 20 mM Tris pH 8.0 and 10% glycerol (w/w) at 4.8 µM, too. The solutions were filtered through a series 0.22 µm membrane and 1 MDa Vivaspin filters with Polyethersulfone membrane (Sartorius, Germany). Higher protein concentrations were obtained with Vivaspin concentrators with Polyethersulfone membrane and 10 kDa molecular weight cut-offs (Sartorius, Germany). Protein concentration for each experiment was determined by the area under a peak from the corresponding High Performance Liquid Chromatography (HPLC) chromatogram. All chemicals were obtained from Sigma unless specified otherwise. Bovine serum albumin (BSA) was purchased from Sigma and dissolved in buffer 50 mM sodium phosphate buffer.

### Fluorescence correlation spectroscopy

Aliquots of naïve HSP60 and GroEL were labeled with TFP Alexa-488 in a 0.1 M sodium-bicarbonate reaction buffer at pH 9.0. To remove the un-reacted probe molecules, the samples were passed through PD MID TRAP G-25 columns and, eventually, separated via HPLC as described in the following paragraph. In the latter step we also measured the protein concentration (111 nM) and the efficiency of labelling (ca. 2 Alexa mols/HSP60 mol). The samples were diluted 1∶2 many times to reach sub-nanomolar concentrations.

The samples were placed in 30 µL wells, previously washed with BSA solution to avoid protein adsorption, for Fluorescence correlation spectroscopy (FCS) measurements on a Hamamatsu C1943 instrument. The excitation wavelength is at 473 nm, while the laser power was kept fixed for all measurements. The experiments were performed at 23°C.

Each measurement consisted of 30 repetitions of 3 second-long acquisitions. The repetitions were used to estimate the statistical error on each point of the correlation function. The latter is computed via software in real time as the time autocorrelation of the fluorescence intensity fluctuations. It contains information on the fluorophore concentration and diffusion. If the convolution of the illuminated volume and the confocal detected region can be described as a Gaussian with an asymmetry ratio of κ, then the correlation function for a single species is
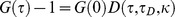
where 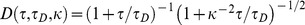


with the diffusion time *τ_D_* inversely proportional to the diffusion coefficient.

To take into account for the small fraction of free probe molecules still present in the samples we fitted the data with the sum of two *D* terms, of which one has its the diffusion time *τ_D_* equal to that measured in a pure 10 nM Alexa-488 solution.

### Dynamic and static light scattering

The samples were placed into a dust-free quartz cell without further filtering and kept at 4 °C and 20 °C in the thermostatic cell compartment of a Brookhaven Instruments BI200-SM goniometer. The temperature was controlled within 0.1°C using a thermostatic recirculating bath. The light scattered intensity and its autocorrelation function were measured at θ = 90° by using a Brookhaven BI-9000 correlator and a 50 mW He–Ne laser tuned at a wavelength λ = 632.8 nm.

Due to their Brownian motion, particles moving in solution give rise to fluctuations in the intensity of the scattered light. The autocorrelator measures the homodyne intensity–intensity correlation function that, for a Gaussian distribution of the intensity profile of the scattered light, is related to the electric field correlation function:
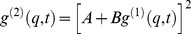
where *A* and *B* are the experimental baseline and the optical constant, respectively. For polydisperse particles, *g^(1)^(q,t)* is given by: 
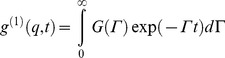



Here, *G*(Γ) is the normalized number distribution function for the decay constant *Γ = q^2^D_T_*, where *q = (4πn/λ)sin(ϑ/2)* is the scattering vector defining the spatial resolution with n, the solvent refractive index and *D_T_*, the translational diffusion coefficient. The hydrodynamic diameter *D_H_* is calculated from *D_T_* through the Stokes–Einstein relationship:
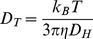
where *k_B_* is the Boltzmann constant, *T* is the absolute temperature, and *η* is the solvent viscosity. Number-weighted distribution functions *P_N_* of the z-average hydrodynamic diameter *D_H_* were obtained by the analysis of the intensity autocorrelation functions were analyzed by means of a CONTIN-likejavascript:void(0); smoothing-constrained regularization method [44].

The scattered intensity *I*(*q*) is given in terms of the Rayleigh ratio *I*(*q*)/*I*_s_ *r* ^2^/*V*_s_, where *I*_s_ is the intensity of the laser source, *V*_s_ is the scattering volume, and *r* is the distance of the detector from the sample. Absolute values for scattered intensity were corrected for the scattering from buffer alone and normalized by the intensity of a toluene standard, whose Rayleigh ratio was taken as 14×10^−6^ cm^−1^ at 632.8 nm. Absolute Rayleigh ratio *R*(*q*) is related to the weight averaged molecular mass *M*_w_ of particles by the relation: *R*(*q*) =  *KcM*_w_ *P*(*q*), with the instrumental factor *K* =  4π^2^ *ñ* ^2^(d*ñ*/d*c*)^2^λ_0_^−4^ *N*_A_^−1^, where *c* is the mass concentration, *P*(*q*) is the *z*-averaged form factor, *ñ* is the medium refractive index, λ_0_ is the incident wavelength, and *N*_A_ is the Avogadro's number [45]. We calculated the average molecular mass *M*_w_ by taking (d*ñ*/d*c*)  = 0.18 cm^3^ g^−1^, and *P*(*q*) = 1. The form factor is related to the average shape and size of scatterers. However, it is equal to 1 when the size of solutes is much smaller than *q* ^−1^ [46].

### Blue native polyacrylamide gel electrophoresis

Native gel electrophoresis was performed using NativePAGE Bis-Tris Gels according to the manufacturer's instructions (Invitrogen, Carlsbad, CA, USA). Hsp60 and GroEL protein samples were diluted in NativePAGE Sample Buffer (1x) containing 1% digitonin and 0.5% n-dodecyl-β-D-maltoside (DDM) detergent solutions at pH 7.2. Samples and molecular weight marker (NativeMark Unstained Protein Standard-Invitrogen) were loaded on precasted 4–16% Novex NativePAGE Bis-Tris gels that resolve proteins in the molecular weight range of 15–1,000 kDa. lectrophoresis was performed at 150 V constant voltage for 120 min, using XCell SureLock Mini-Cell (Invitrogen). Gels were stained by Coomassie G-250 staining. Gels were destained in 8% acetic acid until the desired background was obtained and scanned, using Gel Doc XR (Bio-Rad) molecular imager. Protein molecular weights were determined by the Quantity One software (Bio-Rad).

### HPLC system and conditions

Chromatographic separations were performed with an HPLC device (LC-2010 AT Prominence, Shimadzu, Kyoto, Japan), equipped with an UV-Vis photodiode array detector and a 20 µL sample loop. The samples injected at 24 °C were eluted with a flow of 0.5 ml min^−1^ in the sample buffer (Tris - HCl pH 8+10% glycerol 20 mM) degassed by an in-line degasser filter (DGU 20A5). The chromatographic separations were achieved using as size exclusion columns with different separation range: two Shodex 806 and 804 coupled in series. The area of the chromatographic peaks recorded at 280 nm, were normalized and used to determine the concentrations of all samples studied in this work.

### Small Angle X-ray scattering

Small Angle X-ray scattering (SAXS) experiments were performed at the 5.2 beamline of Elettra Synchrotron in Trieste, Italy. Measurements were carried out at 20°C, using a sealed 1 mm diameter glass capillary enclosed within a thermostatic compartment connected to an external circulation bath and a thermal probe for temperature control. The sample-detector distance was set to 2.72 m and the X-ray wavelength λ was 0.154 nm. Because the scattering vector is Q =  4π sin(ϑ/2) λ^−1^ (where ϑ is the scattering angle), the investigated Q-range was 0.06<Q<3.0 nm^−1^. SAXS profiles were recorded on an image plate detector and for each SAXS measurement the acquisition time was 2 minutes. Each sample was measured three times in order to improve the signal to noise ratio, waiting 5 min between each measurement in order to avoid radiation damage. Raw data were radially averaged, considering the beam center from rat tail x-ray diffraction pattern. Both protein solutions and buffers were measured, hence the proteins scattering cross section dΣ/dΩ(Q) was obtained by properly subtracting from the protein solution scattering curves the empty cell and buffer contributions [47]. Data analysis was performed by using the Guinier law [48], according to which the scattering curve, at low Q range, can be approximated as
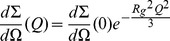



The particle gyration radius R_g_, is linked to sample shape and dimension, and provides an estimation of protein aggregation. Hsp60 was measured at concentrations c = 23.8 µM and at c = 79.5 µM, while GroEL at c = 52.5 µM. The concentrations of all samples were checked before and after SAXS experiments by HPLC chromatograms.

## Results

### Oligomeric structure of naïve Hsp60 at micro-molar concentrations

To gain insight into the naïve Hsp60 oligomeric structure, we investigated the protein properties and multimeric self-assembly equilibria by High Performance Liquid Chromatography (HPLC), dynamic and static light scattering (DLS and SLS, respectively), and blue native PAGE. All these techniques allowed us to explore naïve Hsp60 at micro-molar concentrations in the range 0.8 to 47.6 µM.

We first used HPLC to determine if, similarly to mtHsp60 in the same concentration range [16], [17], naïve Hsp60 occurs as heptameric/tetradecameric oligomers. To determine the influence of the protomer concentration on the protein multimeric state in solution and the contribution of cooperativity to the oligomerization rate, we carried out size exclusion chromatography. In parallel, we did the same with GroEL, which occurs as a tetradecamer (798 kDa) made up of two heptameric rings [49]. In addition, we ran bovine serum albumin (BSA), a protein with molecular weight, 66,5 kDa, very close to that of the naïve Hsp60 monomer.

**Figure 1:**
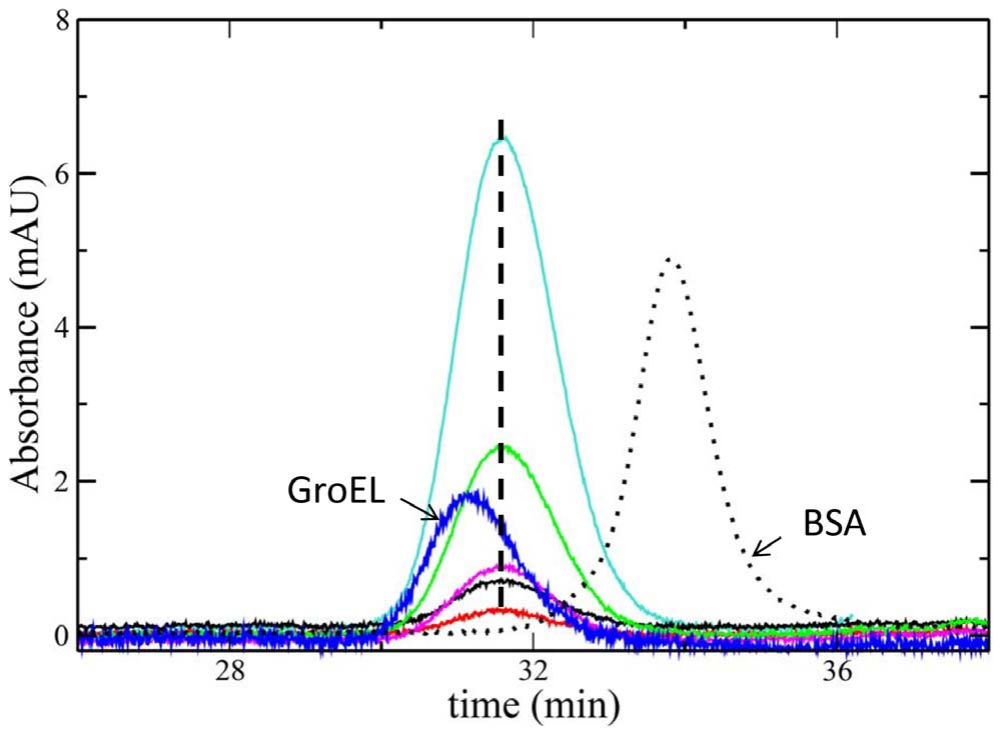
Size exclusion chromatography results. Size exclusion chromatography experiments on the naïve Hsp60 at different concentrations (0.8 µM: red, 1.6 µM: black, 2.2 µM: magenta, 6.4 µM: green, 16.0 µM: turquoise) compared with GroEL at 7.0 µM (blue) and BSA (dotted line). The vertical line drawn across the Hsp60 peak helps to highlight the independency of the retention time from protein concentration. The value of the retention time is consistent with that expected for heptameric and tetradecameric species in equilibrium.

The results shown in Figure 1 revealed that the naïve Hsp60 retention time (R.T.) is, at all the concentrations tested, considerably lower than that of monomer BSA and higher with respect to GroEL tetradecamer. As the molecular weight of BSA monomer is 66,5 kDa [50], very similar to that of Hsp60 (63 kDa), we can safely assume that Hsp60 solution does not include free monomers. On the other hand, the fact that R. T. of Hsp60 is higher than that of the GroEL tetradecamer, suggests an equilibrium between different oligomers. Since the molecular weight of the GroEL monomer (57 kDa) is lower than that of naïve Hsp60 (63 kDa), the elution peak observed for the human chaperonin cannot be attributed to the presence of tetradecamers (which should have manifested themselves with a lower retention time with respect to GroEL), but it rather suggests an equilibrium between heptamers and tetradecamers.

**Figure 2:**
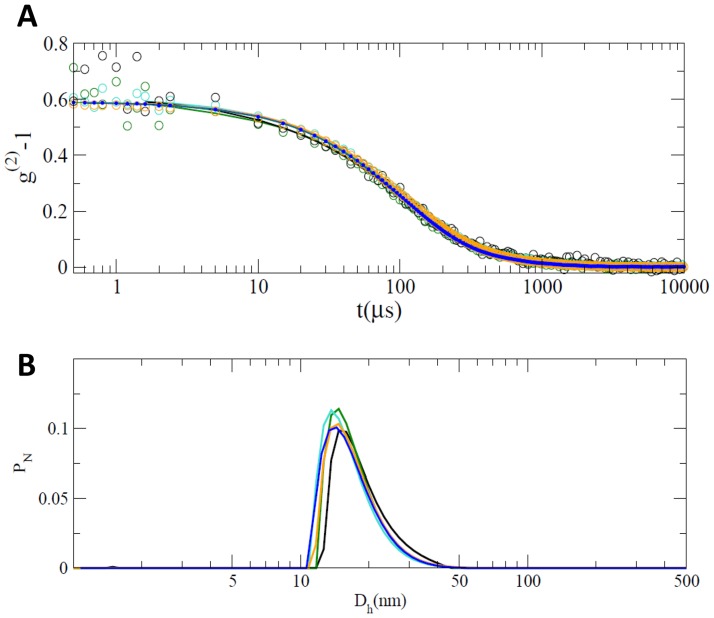
Dynamic light scattering characterization. Dynamic light scattering characterization of the naïve Hsp60 at different concentrations (0.8 µM: black, 6.4 µM: green, 16.0 µM: turquoise, 27.0 µM: orange) compared with GroEL at 7.0 µM (blue). (A) Normalized intensity autocorrelation functions g^(2)^(t). (B) Number-weighted distribution functions P_N_ of the *z*-average hydrodynamic diameter D_H_ obtained by the analysis of the autocorrelation functions. At each concentration, the hydrodynamic diameter of Hsp60 is always compatible with that of heptamer/tetradecamer species.

The absence of monomeric forms of naïve Hsp60 resulting from heptamer or tetradecamer destabilization was confirmed by DLS, a non-invasive technique, widely used to characterize the hydrodynamic radius of proteins and protein aggregates or oligomers in solution [51]–[54]. Again, measurements were performed by comparing naïve Hsp60 at various concentrations and GroEL at a fixed concentration. Figure 2 shows the autocorrelation functions of the scattered light intensity at q = 18.7 µm^−1^ (A), and the hydrodynamic diameter distributions, represented as number-weighted distributions (B). The autocorrelation functions were superimposable for all naïve Hsp60 concentrations tested and GroEL. Moreover, results from number-weighted distributions showed that, for each Hsp60 concentration, the most populated species had hydrodynamic diameters in the range 12–25 nm, consistent with the dimensions of heptamers and tetradecamers. The number distribution of GroEL size showed a peak in the same region. The number of larger aggregates, responsible for other decays present in autocorrelation functions, were negligible for all the samples under analysis. Although DLS did not distinguish between heptamers and tetradecamers, it excluded the presence of monomers for all of the Hsp60 concentrations tested.

**Figure 3:**
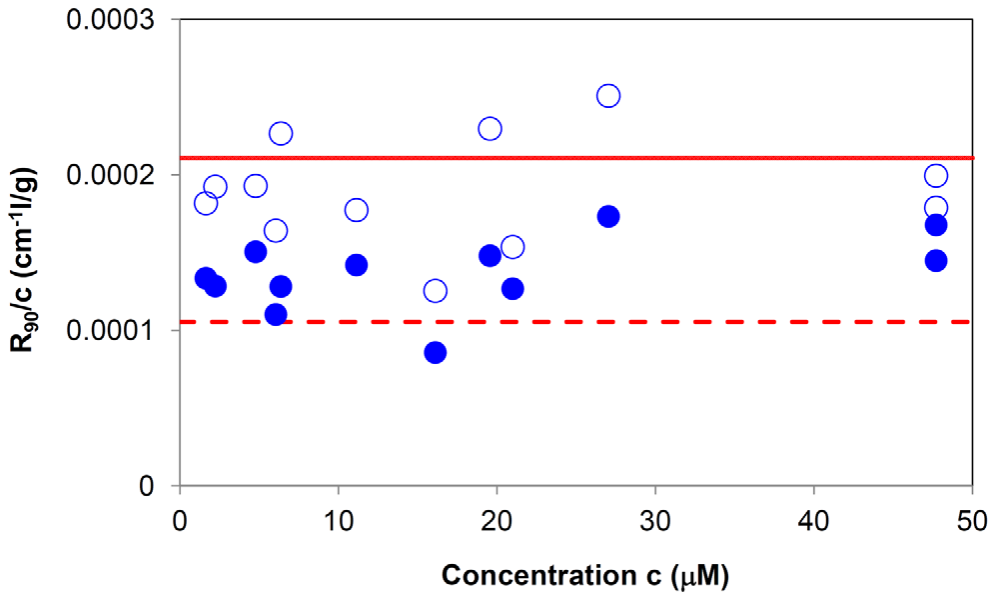
Static light scattering characterization. Scattered light intensities from solutions of naïve Hsp60 protein at different concentrations, expressed in terms of the Rayleigh ratio *R_90°_* at 18.7 µm^−1^ normalized by the concentration values *c*. Together with the total scattered intensity (empty circles), the intensity contribution by species with diameter size lower than 70 nm (filled circles) is also reported. The lines represent the dependence of *R_90°_/c* on concentration predicted for Hsp60 protein totally assembled in tetradecamers (solid) or heptamers (dashed). The experimental values of intensity scattered by Hsp60 species always fall between those predicted for totally tetradecameric or heptameric populations.

To gain further insight into the protein oligomeric equilibrium, naïve Hsp60 at various concentrations was also characterized by SLS. Figure 3 displays the intensity scattered at *q* = 18.7 µm^−1^ in a concentration range from 1.6 to 47.6 µM, in terms of the Rayleigh ratio R at 90° scattering angle, R_90_, divided by the protein concentration. In the same graph, the ideal straight lines for the tetradecameric (882 kDa) and heptameric (441 kDa) structures are shown. Data relative to naïve Hsp60 fell in the intermediate region between the two ideal lines and just above the line for tetradecamers. In the same graph, we show the intensity values of only the species with hydrodynamic radius compatible with naïve Hsp60 oligomers, excluding higher-size molecular aggregates. The data show that, in this case, the R_90_/c values fell between the intensity scattered by an ideal heptamer population and another constituted of only tetradecamers, suggesting a heptamer/tetradecamer equilibrium throughout the concentrations range tested.

These results were confirmed by repeating HPLC and scattering experiments at various times (1, 4, and 7 days from the moment stock samples were thawed just before testing) and temperatures (from 4 to 20°C) (data not shown). These results revealed that the naïve Hsp60-oligomer stability requires physicochemical conditions different from those required by mtHsp60. In fact, mtHsp60 proved to be highly unstable since it rapidly dissociated into monomers, with the dissociation being enhanced by lowering the assay temperature from 25 down to 4°C, and by diluting the samples down to 3.3 µM [17], [24].

**Figure 4:**
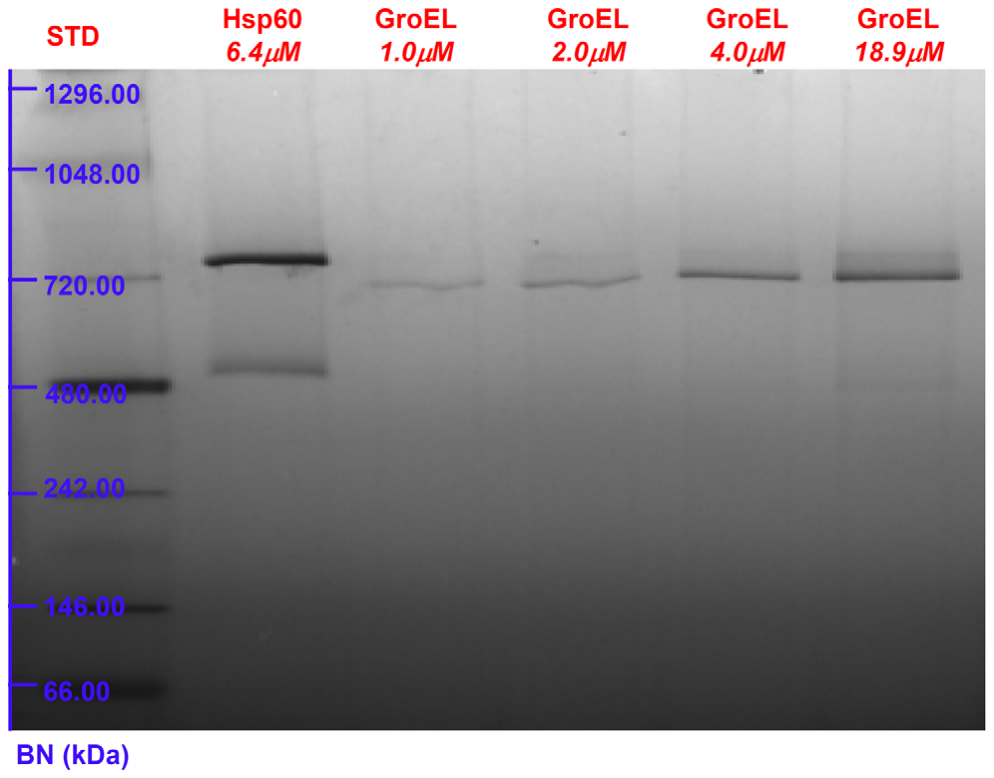
Blue Native Polyacrylamide Gel Electrophoresis: comparison of Hsp60 with GroEL. Blue Native PAGE (4–16%) image of GroEL in the concentration range of 1.0÷18.9 µM and naïve Hsp60 protein at 6.4 µM. GroEL protein gives rise to a single band corresponding to the tetradecameric structure. On the contrary, naïve Hsp60 protein, at the intermediate concentration of 6.4 µM, presents two bands that can be attributed to tetradecamers and heptamers, respectively.

**Figure 5:**
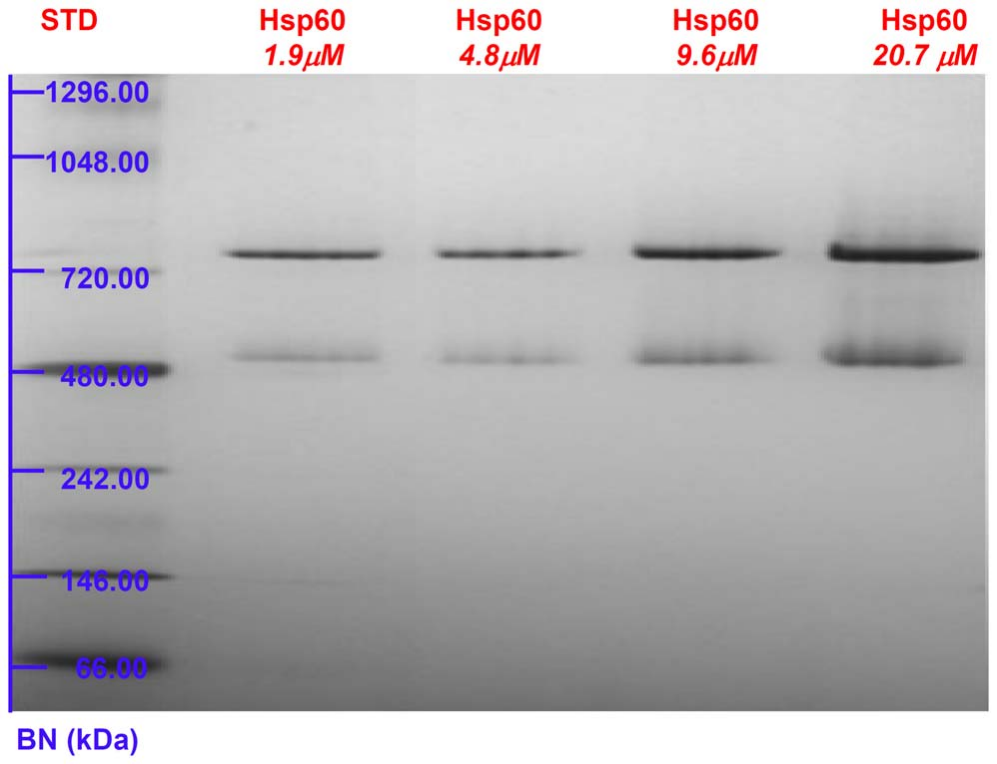
Blue Native Polyacrylamide Gel Electrophoresis: Hsp60 at various concentrations. Blue Native PAGE (4–16%) image of naïve Hsp60 in the concentration range 1.9–4.8 µM. The pattern reveals that the protein exists in two oligomeric forms independently of the concentration.

A deeper analysis of naïve Hsp60 oligomeric equilibria comes from the blue native (BN) PAGE experiments. BN-PAGE is extensively used for the analysis of non-dissociated protein complexes to determine molecular mass, composition and oligomeric state [55]. Results showed that, while GroEL at different concentrations in the 1.0 to 18.9 µM range gives rise to a unique single band corresponding to the tetradecameric structure, the naïve Hsp60 is resolved into two bands (Figures 4 and 5). Based on the measured molecular masses, the two bands can be attributed to the heptamers and tetradecamers, respectively with the band pertaining to the latter being more intense than that pertaining to the heptamers (Figure 4). This pattern differs from that of mitochondrial Hsp60, which is mainly heptamer with a minor fraction of tetradecamers and monomers [17]. In order to evaluate the impact of protein concentration on the heptamers/tetradecamers equilibrium, the naïve Hsp60 oligomerization state was investigated by BN-PAGE at various concentrations, from 1.9 µM to 20.7 µM, The gel image showed that the protein existed in a dual oligomeric form at all concentrations tested (Figure 5).

### Oligomeric structure of naïve Hsp60 at high micro-molar concentrations

**Figure 6:**
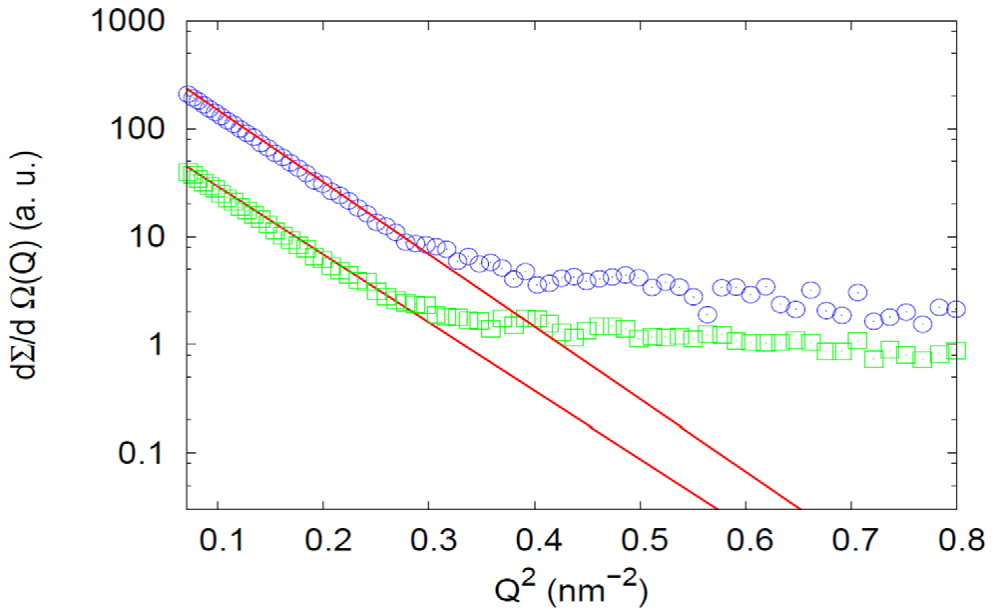
SAXS for GroEL and Hsp60: Guinier approach. SAXS profiles of naïve Hsp60 at 23.8 (green) and 79.5 µM (blue). Experimental curves are scaled for the sake of clarity. Continuous lines correspond to the theoretical fitting obtained by the Guinier approach, as reported in Material and Methods, SAXS. The gyration radius extrapolated by fitting procedure suggests, at both concentrations, the simultaneous presence of oligomeric structures in equilibrium.

To gain further insight into the naïve Hsp60 oligomeric status and compare it to GroEL, we did SAXS measurements at two protein concentrations [23.8 µM and 79.5 µM]. The results showed differences between naïve Hsp60 and GroEL structures in solution. In Figure 6, the low Q-range curves relative to Hsp60 at two different concentrations are displayed, together with the theoretical fitting curves. Although the shapes of the SAXS curves were not dramatically different, the average gyration radii obtained by the Guinier's approach were not exactly equivalent. In fact, at the lower protein concentration (c = 23.8 µM), R_g_ was 6.58±0.08 nm, while at the higher protein concentration (c = 79.5 µM), R was 6.80±0.08 nm. The latter value is comparable with the estimated gyration radius of GroEL (R_g_ = 6.78±0.08 nm) in agreement with the estimation of Arai et al. [56]. These findings suggest the simultaneous presence of oligomers (tetradecamers/heptamers) in equilibrium. At increasing protein concentrations, the higher molecular weight populations seemed favored; namely, the tetradecameric structure predominated at c = 79.5 µM, whereas a mixture of tetradecamers and heptamers occurred at c = 23.8 µM. This result, although expected for a protein oligomerization process, would indicate a difference between the naïve Hsp60 oligomers and their mtHsp60 counterparts since the latter, that are mainly heptamers, form double rings, i.e., tetradecamers, only in the presence of ATP or Hsp10 [16], [17].

**Figure 7:**
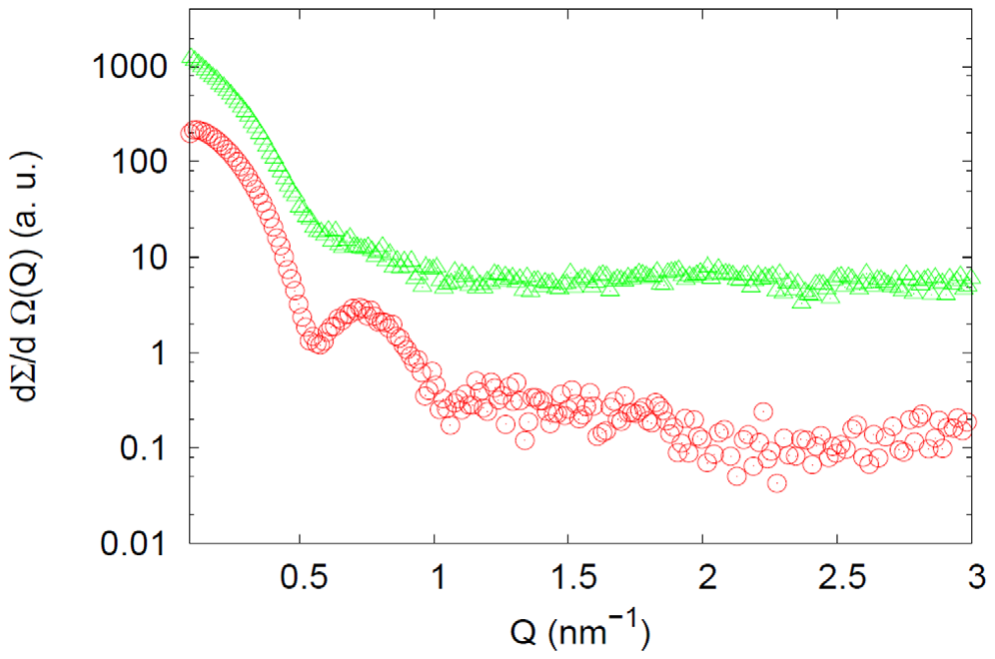
SAXS profiles of GroEL and Hsp60. SAXS profiles of GroEL (red) and naïve Hsp60 (green). GroEL is at c = 52.5 µM, while Hsp60 is at c = 79.5 µM. Experimental curves are scaled for the sake of clarity. SAXS spectrum for Hsp60 is quite different from that of GroEL, which corresponds to a more well-defined structure.

The comparison between GroEL and Hsp60 SAXS spectra deserves further attention. While according to the simple Guinier approximation the average gyration radii of the two proteins at moderate concentrations are comparable, the features of the differential cross sections show remarkable differences, as it is evident from data in Figure 7. The SAXS curve corresponding to native GroEL is characteristic of the protein's well defined structure and is comparable with other results in the literature [56], [57].

On the other hand, the Hsp60 SAXS spectrum is quite different, from that of GroEL, and it could be explained by considering that Hsp60 tetradecamers can show remarkable differences in comparison to GroEL tetradecamers, starting from the monomer and continuing up to the more complex structures as it is suggested by their different ability to function with co-chaperonins from different sources [25], [58]. Also, making a point of Hsp60 retention times (Fig. 1) SLS (Fig. 3) and electrophoresis (Fig. 4, 5) results, it has to be considered the simultaneous presence of tetramers and heptamers in Hsp60 solution, that gives rise to a SAXS profile which is due to the different oligomers in solution. Hence, SAXS data confirm that GroEL and Hsp60 tetradecameric structures in solution are different, but due to the simultaneous presence of different oligomers in Hsp60 solution it is not possible to obtain their shape reconstruction.

### Oligomeric structure of naïve Hsp60 at nano-molar concentrations

**Figure 8:**
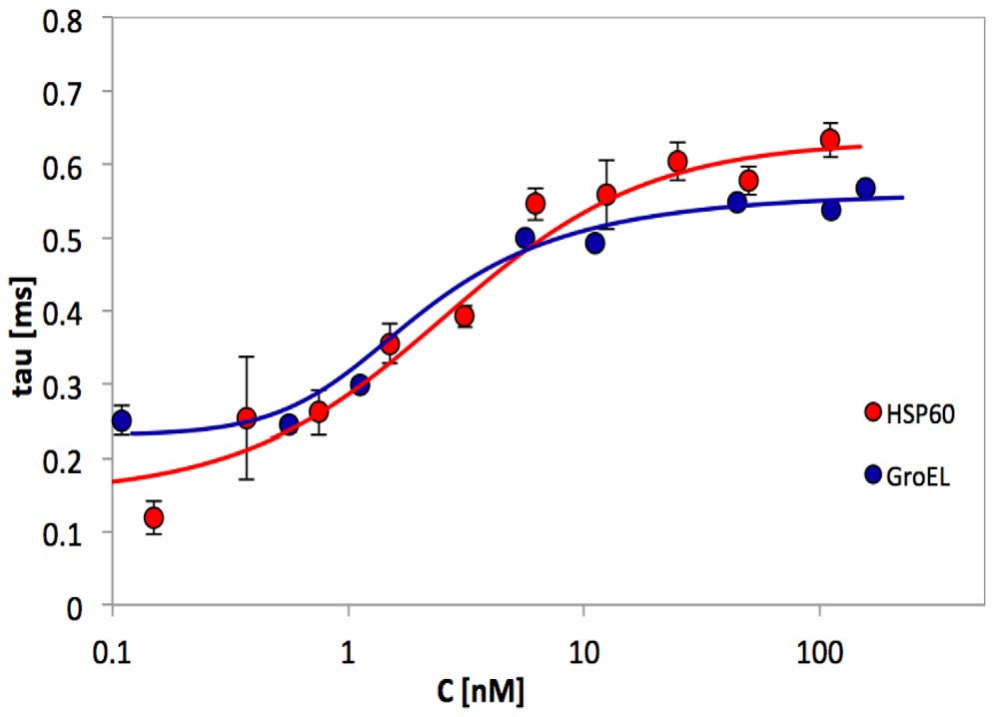
Fluorescence correlation spectroscopy results. Diffusion times measured by FCS as a function of monomer concentration: GroEL (red), naïve Hsp60 (blue). Continuous lines correspond to interpolations of the experimental data to sigmoid functions and are drawn as a guide for the eye. Diffusion times, both for Hsp60 and GroEL remain constant with lowering the concentration down to 10 nM, thus indicating a high stability of the protein oligomeric complexes.

In order to understand if the interactions between monomers typical of Hsp60 naïve oligomers are permanent or transient protein-protein interactions (PPI) [59], we decided to investigate the naïve Hsp60 oligomeric structure at nano-molar concentrations [from 10 to 100 nM], namely, levels that, to our knowledge, had not been examined before, and which would provide information on the stability of oligomers at very low concentrations. In order to do this, we used Fluorescence Correlation Spectroscopy (FCS), a method that, by correlation analysis of the protein-bound fluorophore time-dependent fluorescence, allows determining diffusion coefficients and multimerism of proteins. FCS tests can be carried out at very low concentrations with high sensitivity and even at the single-molecule level [60]. Figure 8 shows naïve Hsp60 (labeled with Alexa TFP Fluor dye) average diffusion times, resulting from fluorescence autocorrelation function analysis, *versus* protein concentration and in comparison to its bacterial homolog GroEL. Data were interpolated to a sigmoid function as a guide for the eye.

At the highest concentration, the average diffusion time of naïve Hsp60 was compatible with that of objects with a hydrodynamic radius of ca. 7.5 nm while in the case of GroEL the radius was slightly smaller (7.0 nm).

These analyses revealed that diffusion times of naïve Hsp60 and GroEL oligomers remain approximately constant while the protein concentration is lowered from 100 nM down to 10 nM. This indicates that, similarly to GroEL, Hsp60 heptamers and tetradecamers are very stable even under destabilizing conditions such as those resulting from the fluorescent probe labeling. A variety of conditions destabilize GroEL tetradecamers, favoring dissociation to monomers, such as moderate urea concentration [61], high hydrostatic pressure [62], presence of nucleotides and adsorption onto ion-exchange resins [63], and replacement of Lysine 3 at the N-terminus [64]. The GroEL N-terminal region is located in the subunit interface and is involved in links that stabilize the protein's quaternary structure. Moreover, mutations in the region encompassing the first 4 amino acids of the mtHsp60 N-terminal region destabilize the protein oligomeric state causing its disassembly at low protein concentrations [17]. It is noteworthy that the amine-reactive Alexa Fluor dye reacts with non-protonated aliphatic amine groups, especially lysine amino groups. Lysine at position 28 (position 3 in the sequence Tyr-Ala-**Lys**-Asp-Val-Lys-Phe-Gly-Ala) is a highly conserved amino acid present in the human chaperonin Hsp60 [64]. The lower size observed for GroEL with respect to Hsp60 from their diffusion times at the highest concentration might be the result of a different destabilizing effect of the dye on the two proteins.

FCS data show that the progressive decrease in concentration down to 10 nM does not cause dissociation of the naïve Hsp60 oligomers, even if their oligomerizing potential is weakened by the labeling procedure. The reduction in diffusion times that we observed below the concentration of 10 nM for both GroEL and naïve Hsp60 and compatible with the formation of monomers, could be attributed to the existence of a very low threshold concentration below which monomers do occur, but it cannot be excluded that it is the consequence of the destabilization effect caused by the protein-fluorophore binding.

## Discussion

The purpose of our work was to determine the structural arrangement in solution of naïve Hsp60, i.e., whether it forms heptamers and tetradecamers as mtHsp60 and GroEL do, and the impact of protein concentration on oligomer stability. We investigated three ranges of concentrations using various complementary biophysical methods.

Despite the considerable number of reports dealing with the possible pathogenic significance of Hsp60-concentration increase in the cytosol (see for review Ref. [65]), the structural characteristics of the cytosolic Hsp60 have not been elucidated. For example, it is not yet known if the Hsp60 molecules that accumulate in the cytosol still bear the MIS (naïve) or it is mtHsp60 exported by mitochondria, or if both types of molecules co-exist. MtHsp60 had been well characterized in previous studies that had shown the occurrence of an equilibrium between monomeric/heptameric and tetradecameric forms, strongly influenced by protein concentration, temperature, and presence of cofactors (ATP and Hsp10) [16]. No or little information is available about naïve Hsp60, and we decided to focus on it.

If Hsp60 still bearing the MIS is the one that accumulates in the cytosol in specific pathological conditions, the data in this report should help future research aiming at elucidating the functions and mode of action of naïve Hsp60 in pathogenesis, especially in those diseases in which the chaperonin increases as the pathological manifestations progress and become severe.

Therefore, we decided to carry out a structural comparison between recombinant naïve Hsp60 and its prokaryotic homolog GroEL. We used as a baseline GroEL instead of a recombinant Hsp60 lacking the MIS because the former had been extensively studied and we could benefit from the data already available for comparative analyses. GroEL, under physiological conditions assembles into a stable complex made of two stacked heptameric rings, which is considered the functional chaperoning structure [66].

By using FCS, we explored concentration values at the nano-molar level, whereas the characteristic hydrodynamic sizes of the oligomeric complex for GroEL and naïve Hsp60 were investigated by DLS and SLS in solution at the micro-molar level. Lastly, using SAXS, we explored Hsp60 oligomeric structures at still higher micro-molar concentrations.

We found that naïve Hsp60 can form heptamers and tetradecamers that are stable over a wide range of concentrations, thus indicating the occurrence of strong protein-protein interactions characterizing the oligomeric complexes [59].

Our experiments in vitro did not take into account the complex cellular environment, including molecular chaperones, such as Hsp70 and Hsp90 that, in the cytosol, deliver the proteins to the mitochondrial import system [7], [8]. However, our data unveiled the structure of naïve Hsp60 in solution for the first time and should stimulate investigations on the structure/function relationships of cytosolic Hsp60 in normal and pathological situations. In fact, the demonstration that the naïve Hsp60 is able to form oligomeric species that, for mtHsp60 and its bacterial homolog GroEL, are considered the functional form of the chaperonins, could corroborate that Hsp60 with MIS is not an aberrant protein that failed to enter mitochondria, but it could accumulate in the cytosol and be involved in physiological functions as oligomer. If it also participates in cellular functions as monomer, it may be assumed that it occurs, and functions, at concentrations lower than those we tested, namely lower than that necessary for oligomerization. Alternatively, one can imagine that a mechanism exists for blocking oligomerization of a fraction of the newly synthesized Hsp60, for example post-translational modification, occurring as the peptide is emerging from the ribosome or immediately thereafter. However, other mechanisms cannot be ruled out, including the possibility that while some types of post-translational modification impede oligomerization other types favor it, depending on the cell's physiological or pathological state, cell's needs, and intracellular conditions. No doubt, investigations on the structure and functions of cytosolic Hsp60 should help to understand its chaperonopathies, namely, those pathological conditions in which the chaperonin plays an etiologic-pathogenic role. Furthermore, elucidation of the Hsp60 structure that functions in the cytosol should greatly facilitate the rationalization of the model to be used for the computer-aided design of Hsp60 inhibitory compounds [67].

## Supporting Information

Figure S1 **ATPase Activity of recombinant proteins.** Naïve Hsp60 ATPase and GroEL ATPase activity. Lane 1 (buffer control); Lane 2: ATPase activity of recombinant naïve Hsp60 (250 ng); Lane 3: ATPase activity of naïve Hsp60 (500 ng); Lane 4: ATPase activity of recombinant GroEL (250 ng); Lane 5: ATPase activity of recombinant GroEL (500 ng). Panel A, 3 hrs of TLC exposition; Panel B, 24 hrs of TLC exposition. The proteins used for all the experiments have been able to hydrolyze ATP.
(TIF)
