# Supplementary figures and images for: Human Hsp60 with Its Mitochondrial Import Signal Occurs in Solution as Heptamers and Tetradecamers Remarkably Stable over a Wide Range of Concentrations
Source: PLoS One. 2014 May 15;9(5):e97657. doi: 10.1371/journal.pone.0097657 (PMC4022648; doi:10.1371/journal.pone.0097657)

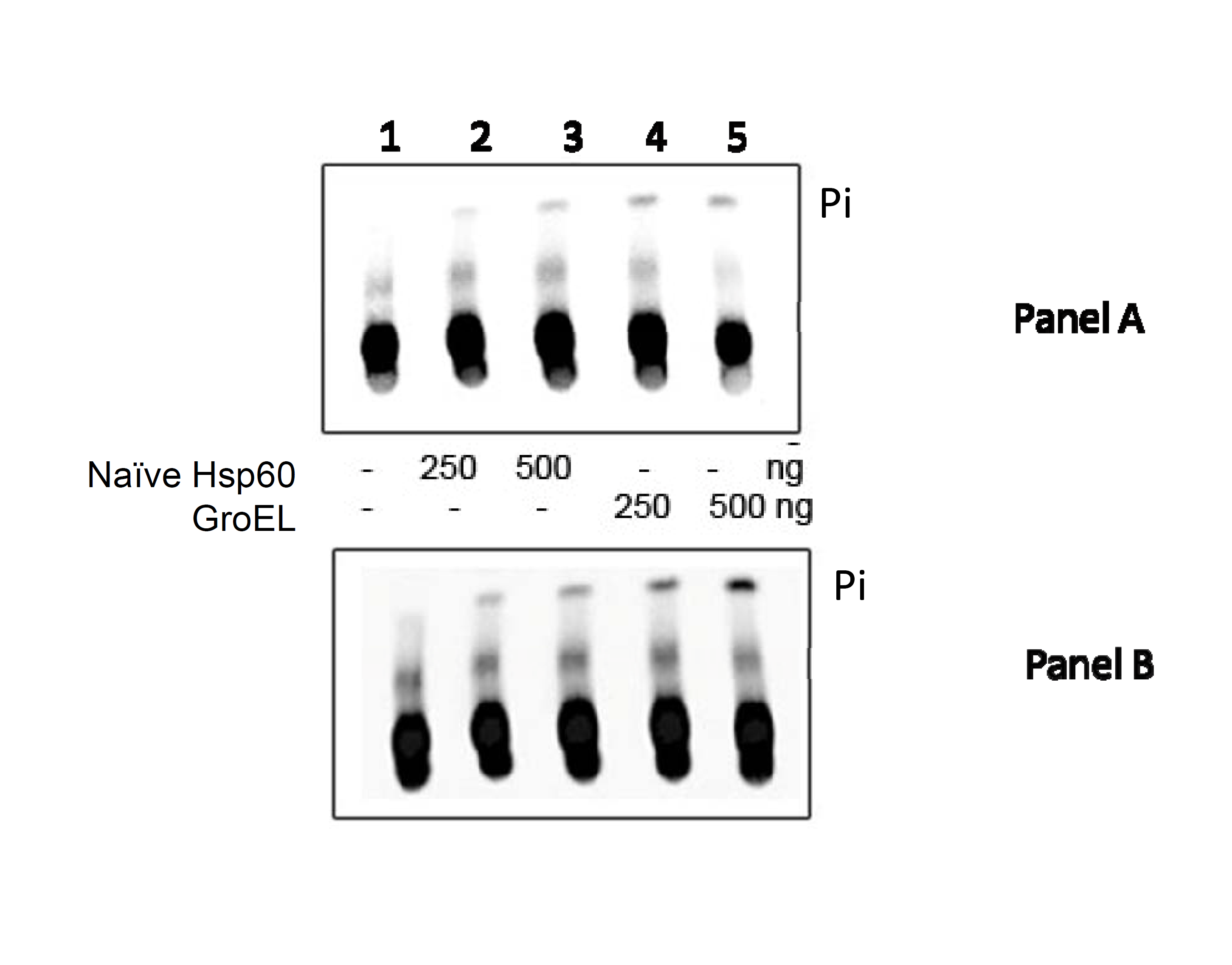

Supplement: Figure S1 — ATPase Activity of recombinant proteins. Naïve Hsp60 ATPase and GroEL ATPase activity. Lane 1 (buffer control); Lane 2: ATPase activity of recombinant naïve Hsp60 (250 ng); Lane 3: ATPase activity of naïve Hsp60 (500 ng); Lane 4: ATPase activity of recombinant GroEL (250 ng); Lane 5: ATPase activity of recombinant GroEL (500 ng). Panel A, 3 hrs of TLC exposition; Panel B, 24 hrs of TLC exposition. The proteins used for all the experiments have been able to hydrolyze ATP. (TIF) [file pone.0097657.s001.tif]
